# Facile Construction of All-Solid-State Z-Scheme g-C_3_N_4_/TiO_2_ Thin Film for the Efficient Visible-Light Degradation of Organic Pollutant

**DOI:** 10.3390/nano10040600

**Published:** 2020-03-25

**Authors:** Wan Zhao, Xiuru Yang, Chunxi Liu, Xiaoxiao Qian, Yanru Wen, Qian Yang, Tao Sun, Wenya Chang, Xin Liu, Zhi Chen

**Affiliations:** 1 College of Materials and Chemistry, China Jiliang University, 258 Xueyuan Street, Xiasha Higher Education District, Hangzhou 310018, China; 17826827277@163.com (W.Z.); yxr15957119921@126.com (X.Y.); 15867130507@163.com (C.L.); qxiao1116@126.com (X.Q.); czmichael@hotmail.com (Y.W.); czmichael@163.com (Q.Y.); 15958003262@163.com (T.S.); 18757556442@163.com (W.C.); 2 College of Standardization, China Jiliang University, 258 Xueyuan Street, Xiasha Higher Education District, Hangzhou 310018, China

**Keywords:** thin-film photocatalyst, environmental control, antibiotic residue removal, g-C_3_N_4_/TiO_2_, all-solid-state Z-scheme heterojunction

## Abstract

The increasing discharge of dyes and antibiotic pollutants in water has brought serious environmental problems. However, it is difficult to remove such pollutants effectively by traditional sewage treatment technologies. Semiconductor photocatalysis is a new environment-friendly technique and is widely used in aqueous pollution control. TiO_2_ is one of the most investigated photocatalysts; however, it still faces the main drawbacks of a poor visible-light response and a low charge-separation efficiency. Moreover, powder photocatalyst is difficult to be recovered, which is another obstacle limiting the practical application. In this article, g-C_3_N_4_/TiO_2_ heterojunction is simply immobilized on a glass substrate to form an all-solid-state Z-scheme heterojunction. The obtained thin-film photocatalyst was characterized and applied in the visible-light photodegradation of colored rhodamine B and tetracycline hydrochloride. The photocatalytic performance is related to the deposited layers, and the sample with five layers shows the best photocatalytic efficiency. The thin-film photocatalyst is easy to be recovered with stability. The active component responsible for the photodegradation is identified and a Z-scheme mechanism is proposed.

## 1. Introduction

Water pollution has become particularly serious in recent decades due to the rapid development of industrialization [1]. The photocatalytic technique provides an alternative strategy to solve the above problem since it converts solar energy directly into useful energy for degrading organic pollutants into harmless components [2]. In 1977, Frank reported [3] the successful degradation of pollutants in water by using TiO_2_ as the photocatalyst, which aroused extensive applications of TiO_2_ in water treatment. In 1987, Mattews et al. [4] found that 34 kinds of organic pollutants in water could be degraded by TiO_2_ under the irradiation of ultraviolet light (UV-light). Until now, TiO_2_ is one of the most popular photocatalysts for environmental protection [5,6].

However, pristine TiO_2_ photocatalyst has two intrinsic defects of a wide bandgap (3.2 eV) and the fast recombination of photogenerated carriers, which limits its widespread development. It is necessary to modify pristine TiO_2_ to simultaneously satisfy the requirements for practical applications of photocatalytic techniques, such as intensive visible-light response, high photogenerated-carriers separation efficiency, good stability, and strong redox capabilities [7,8,9]. g-C_3_N_4_ is a recently emerged nonmetallic photocatalyst with a narrow bandgap (2.7 eV), demonstrating prominent features such as a visible-light response, good chemical stability, and easy synthesis [10,11,12]. Wang et al. [13] reported the efficient water splitting by using g-C_3_N_4_ as the visible-light photocatalyst in 2009. This has evoked great interests, and diverse approaches have been proposed to improve the photocatalytic efficiency. Among these strategies, construction heterojunction has been proven as one of the effective strategies [14]. In particular, TiO_2_ and g-C_3_N_4_ can form an all-solid-state Z-scheme heterojunction due to their matched conduction band (CB) and valence band (VB) positions, which can not only overcome the intrinsic defects of pristine photocatalyst but can also maintain the strong redox ability [12,15]. Additionally, the powder photocatalyst is difficult to be recovered from the solution for further use, which is another obstacle restraining the wide application of the photocatalytic technique in water treatment [16,17,18]. Intensive efforts have been made to immobilize the powder photocatalyst on substrates such as aerogels or glass [19,20].

Herein, all-solid-state Z-scheme g-C_3_N_4_/TiO_2_ thin-film photocatalysts were successfully constructed by using the common glass as the substrate from the simple sol-gel and spin coating process. The obtained thin-film photocatalysts show good photodegradation performances for the treatment of colored Rh B and colorless tetracycline hydrochloride under visible light, which are closely related to the coating layers. The active reagent is identified and a Z-scheme mechanism is proposed.

## 2. Experiment

### 2.1. Materials

All the chemicals were purchased from commercial suppliers and were used directly. Absolute ethanol (CH_5_OH), isopropanol (C_3_H_8_O), glacial acetic acid (CH_3_COOH), butyl titanate (C_16_H_36_O_4_Ti), and tetracycline hydrochloride (C_22_H_24_N_2_O_8_·HCl, TC-HCl) were obtained from Shanghai Aladdin Biochemical Technology Co. Ltd. (Shanghai, China). Melamine (C_3_N_3_(NH_2_)_3_) and tert butyl alcohol ((CH_3_)_3_COH, TBA) were bought from Wuxi Prospect Chemical Reagent Co. Ltd. (Wuxi, China). Benzoquinone (C_6_H_4_O_2_, BQ) and ethylene diamine tetraacetic acid (C_10_H_16_N_2_O_8_, EDTA) were obtained from Shanghai Macklin Biochemical Technology Co. Ltd. (Shanghai, China). Rhodamine B (C_28_H_31_ClN_2_O_3_, RB) was purchased from Tianjin Kemio Chemical Reagent Co. Ltd. (Tianjin, China).

### 2.2. Preparation

**Preparation of g-C_3_N_4_ powder.** The preparation of g-C_3_N_4_ was operated according to the previous report [12]. Typically, 10.0 g of melamine was put into an alumina crucible and heated at 523 °C with a ramp rate of 2 °C/min in a muffle furnace under air atmosphere. Then, the solid was cooled down to room temperature after keeping at 523 °C for 4 h and ground to get g-C_3_N_4_ power product.

**Preparation of TiO_2_ sol.** The preparation TiO_2_ sol was similar to the former publication [21]. Generally, it can be specifically divided into the following three steps: (1) 60 mL of absolute ethanol and 20 mL of butyl titanate were successively added into a 250 mL beaker at room temperature, and the mixture was stirred for 30 min to obtain a pale yellow transparent solution (recorded as beaker A); (2) 80 mL absolute ethanol, 20 mL deionized water, and 32 mL glacial acetic acid were successively put into 250 mL beakers at room temperature and stirred continuously with the magnetic force for 30 min, which was defined as beaker B. (3)The solution in beaker B was dropped into beaker A at the rate of 2 drops per second, and the solution was kept constantly stirring for 2h to obtain the sol. The molar ratios of the reagent for the synthesis of TiO_2_ sol were set as follows: absolute ethanol: butyl titanate: deionized water: glacial acetic acid = 40:1:20:8.83. The prepared sol was aged at room temperature for three days and underwent the alcoholysis–hydrolysis–polycondensation reaction to form a stable gel. 

**Preparation of g-C_3_N_4_/TiO_2_ thin film.** Typically, (1) the cleaning of the glass substrate would be carried out as follows: the diced glass substrate (20 × 15 × 1 mm^3^) was washed under ultrasonic in isopropanol, deionized water, and anhydrous ethanol for 15 min in sequence. Then, the cleaned glass substrate was dried at 80 °C and put into a sealed container for future use. (2) In total, 24.0, 48.0, 145.0, 241.0 and 334.7 mg of g-C_3_N_4_ powder was added into 20 mL of TiO_2_ sol that had aged for 3 days, respectively. Then, they were constantly kept magnetically stirring for 20 min to obtain g-C_3_N_4_/TiO_2_ powder-sol with its mass ratio of 0.05, 0.1, 0.3, 0.5 and 0.7. (3) The cleaned glass substrate was stuck to the homogenizer, 200 μL g-C_3_N_4_/TiO_2_ pre-formed sol was dipped on the substrate. The sol was spin-coated on the substrate by a low speed of 500 rpm/min for 10 s and a successive high speed of 4000 rpm/min for 30 s. The coated sample was dried at 80 °C on a heating platform for 5 min, then one layer of film was obtained and the sample was denoted as a 1-layer g-C_3_N_4_/TiO_2_ thin film. Then, 3, 5, and 7 layers of g-C_3_N_4_/TiO_2_ thin film with the mass ratio of 0.5 and 5 layers of pristine TiO_2_ thin film were prepared by a similar spin-coating process and the obtained samples were denoted as 3-layer g-C_3_N_4_(0.5)/TiO_2_, 5-layer g-C_3_N_4_(0.5)/TiO_2_, 7-layer g-C_3_N_4_(0.5)/TiO_2_, 5-layer TiO_2_, respectively. In addition, 5 layers of spin-coated g-C_3_N_4_/TiO_2_ thin films with different g-C_3_N_4_/TiO_2_ mass ratios of 0.05, 0.1, 0.3, 0.7 were prepared in the same way, and the obtained products were named as 5-layer g-C_3_N_4_(0.05)/TiO_2_, 5-layer g-C_3_N_4_(0.1)/TiO_2_, 5-layer g-C_3_N_4_(0.3)/TiO_2_, and 5-layer g-C_3_N_4_(0.7)/TiO_2_, respectively. In addition, g-C_3_N_4_(0.5)/TiO_2_ powder photocatalysts were also prepared from the similar preparation processes of g-C_3_N_4_/TiO_2_ thin-film photocatalyst. All the prepared samples were heated at 450 °C for 2 h with a ramp rate of 2.5 °C/min and automatically cooled down to room temperature.

### 2.3. Characterization

X-ray diffraction (XRD) patterns were acquired from Bruker D2 PHASER X-ray diffractometer (Bruker, Karlsruhe, Germany) with Cu Ka radiation in the range of 10–80°. Fourier transform infrared (FT-IR) spectra were analyzed on Bruker TENSOR 27 Fourier transform infrared spectrometer (Bruker, Karlsruhe, Germany). Scanning electron microscope (SEM) images were recorded on the Hitachi SU 8010 instrument (Hitachi, Tokyo, Japan). The Ultraviolet-visible diffuse reflectance spectrum (UV-vis DRS) was measured on Shimadzu UV-3600 UV-visible using BaSO_4_ as the reference (Shimadzu, Kyoto, Japan). 

### 2.4. Photocatalytic Activity Measurement

The photocatalytic activities of prepared g-C_3_N_4_/TiO_2_ thin films were evaluated by the degradation of colored dye (rhodamine B, Rh B) and colorless antibiotic (tetracycline hydrochloride, TC-HCl) in water. A 300-W Xe lamp with a 420-nm filter was used as the visible light source (≥420 nm). The prepared thin-film photocatalysts were immersed into 50 mL of 5 mg·L^−1^ Rh B solution and 50 mL of 5mg·L^−1^ TC-HCl, and the distance between the light source and the glass substrate was set as 13 cm. The photocatalytic analyses were operated at a constant temperature by a circulating cooling system. Before the light irradiation, the solution with the photocatalyst was magnetically stirred in darkness for 0.5 h to establish adsorption equilibrium. During the light irradiation, 3 mL of the reaction liquid was taken out every 30 min and the solution was analyzed by the UV1600 spectrophotometer to determine the residual concentrate. Then, the measured solution was poured back into the reaction solution to continue the photodegradation.

Antibiotic TC-HCl was employed for stability measurement. Thin-film photocatalyst was immersed into 50 mL TC-HCl (5 mg·L^−1^) and the photodegradation was carried out in the same way as that of Rh B. After a light irradiation of 180 min, the thin-film photocatalyst was taken out and washed with de-ionized water. Then, it was immersed into TC-HCl solution for another cycle of photodegradation, and 5 cycles were run in a similar fashion. As a comparison, a blank experiment without the photocatalyst was done in a similar fashion to study the photo-resistance of Rh B and TC-HCl.

### 2.5. Trapping Experiment

The active species responsible for the photodegradation were identified by the trapping experiments. Diverse trapping agents such as TBA, BQ, EDTA were separately added into the reaction solution (Rh B, 5 mg·L^−1^) and the same operation as the above photodegradation was done. 

## 3. Results and Discussions

### 3.1. Structure, Composition, and Morphology

**Figure 1:**
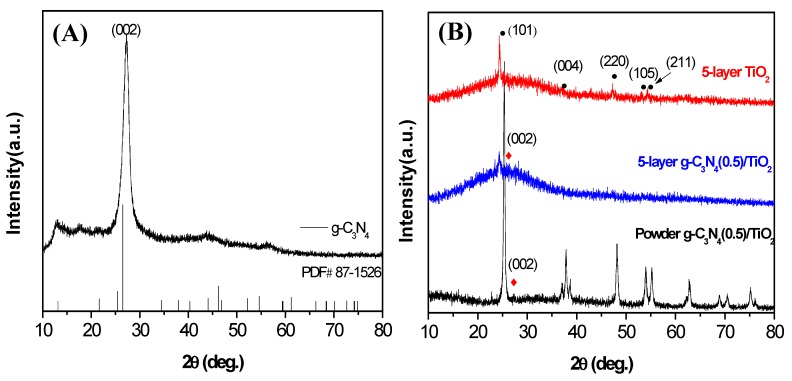
X-ray diffraction (XRD) patterns of (**A**) g-C_3_N_4_ and (**B**) 5-layer TiO_2_, 5-layer g-C_3_N_4_(0.5)/TiO_2_, powder g-C_3_N_4_(0.5)/TiO_2_.

**Figure 2:**
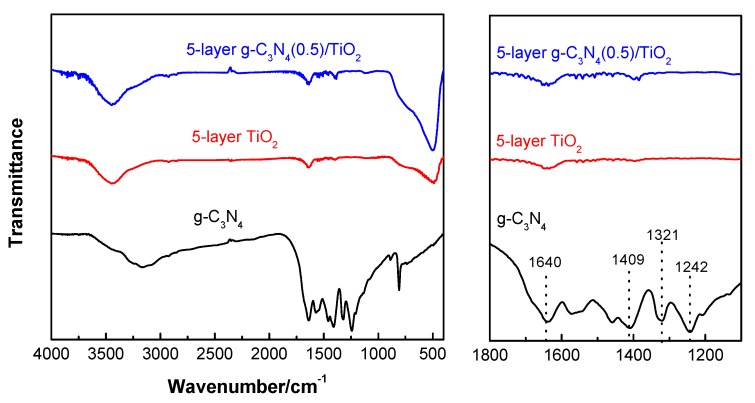
Fourier transform infrared (FT-IR) spectrum of g-C_3_N_4_, 5-layer TiO_2_, 5-layer g-C_3_N_4_(0.5)/TiO_2_.

Figure 1A shows XRD patterns of the synthesized g-C_3_N_4_ and two distinct diffraction peaks at 12.9° and 27.6°, corresponding to the (100) and (002) crystal planes of g-C_3_N_4_ (PDF#87-1526) [22]. The same diffraction bands were also observed on other g-C_3_N_4_-containing samples. As for the TiO_2_-containing photocatalysts (Figure 2B), the characteristic signals of anatase phase TiO_2_ (PDF#21-1272) are presented, and no characteristic peaks associated with the rutile phase are observed in any of the samples. Compared with the 5-layer g-C_3_N_4_(0.5)/TiO_2_, the peak of powder g-C_3_N_4_(0.5)/TiO_2_ has much sharper (101), (004), (200), (105), (211), crystal planes that all corresponds to clear peaks, which indicates the latter has higher crystallinity [23]. However, it is difficult to observe the clear diffraction bands from g-C_3_N_4_, which should be resulted from its low quantity and high dispersion in the thin film.

The FT-IR spectra of the prepared photocatalysts are shown in Figure 2. Characteristic peaks at 1640, 1409, 1321, and 1242 cm^−1^ can be clearly identified on g-C_3_N_4_, which are assigned to the stretching vibration of the aromatic CN heterocycle [24]. Additional peaks at 809 and 3100–3500 cm^−1^ can be attributed to the typical triazine ring vibration and the stretching vibration of N_2_H- and –OH [25]. As for 5-layer TiO_2_, the peaks at about 3400 and 1630 cm^−1^ are assigned to the bending and stretching vibration of OH from the adsorbed water, respectively, which is similar with the previous report [26]. Additionally, the characteristic absorption peak of the TiO_2_ thin film detected in the wavelength range of 450–700 cm^−1^ may be attributed to the stretching vibration of the Ti–O–Ti bond [27]. Only weak characteristic peaks of the g-C_3_N_4_ in 5-layer g-C_3_N_4_(0.5)/TiO_2_ are detected in the FT-IR spectrum due to its low quantity.

**Figure 3:**
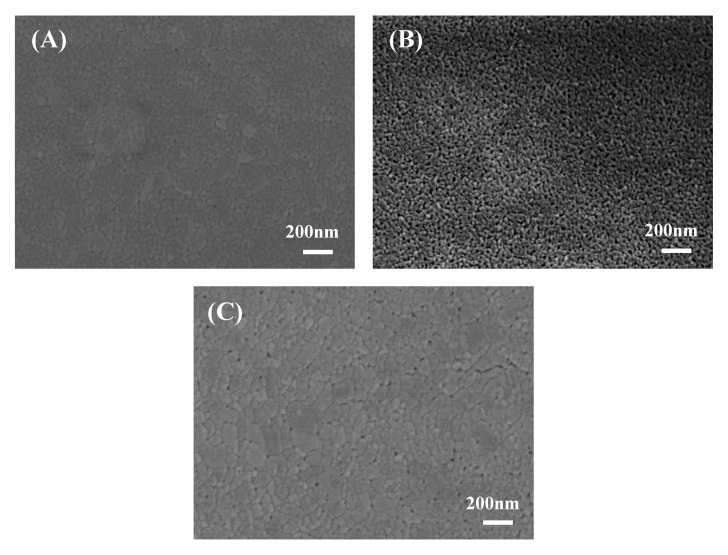
Scanning electron microscopy (SEM) images of different g-C_3_N_4_/TiO_2_ thin-film samples: (**A**) 3-layer g-C_3_N_4_(0.5)/TiO_2_; (**B**) 5-layer g-C_3_N_4_(0.5)/TiO_2_; (**C**) 7-layer g-C_3_N_4_(0.5)/TiO_2._

The surface morphology of the prepared g-C_3_N_4_/TiO_2_ thin-film photocatalyst was studied by SEM, as shown in Figure 3. A flat film can be clearly seen on the glass substrate, as shown in Figure 3A–C. All the films are composed of tightly contacted irregular large and small particles, and pores are also observed at the particle interstice. Along with the increase in film thickness, the surface roughness decreases first and increases afterward. Five-layer g-C_3_N_4_(0.5)/TiO_2_ has the most pores and the smallest particle size with a uniform size distribution, which indicates the presence of the photocatalytic property. 

### 3.2. Optical Property

**Figure 4:**
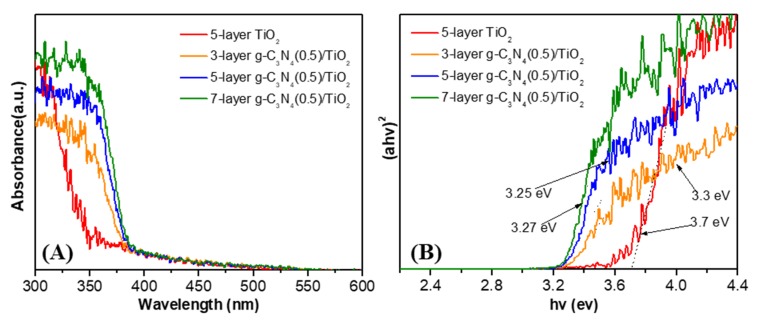
(**A**) Ultraviolet-visible diffuse reflectance spectrum (UV-vis DRS) of 5-layer TiO_2_ thin film and different layers of g-C_3_N_4_(0.5)/TiO_2_; (**B**) The bandgap curve after conversion of the Tauc formula.

Figure 4 shows the UV-vis diffuse spectrum of the prepared samples. As seen in Figure 4A, all the prepared photocatalysts have strong absorption in the ultraviolet region. The absorption band edge moves towards the lower direction with the increase in coating layers, which should come from the increased g-C_3_N_4_. The bandgap is calculated through the Tauc formula and the results are given in Figure 4B [28]. The band gaps of the g-C_3_N_4_-containing photocatalysts are obviously narrower than that of pristine TiO_2_ and become narrower with the increase in coating layers, and the specific band gap data are shown in Table 1. These results indicate that the addition of g-C_3_N_4_ may improve light utilization. 

### 3.3. Photocatalytic Activity

#### 3.3.1. Effects of Film Thickness on the Photocatalytic Performance

**Figure 5:**
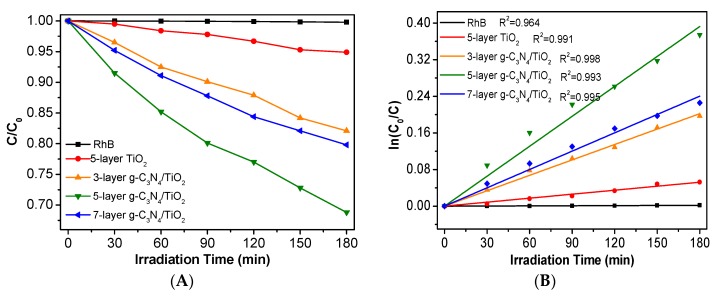
(**A**) Photocatalytic degradation of Rh B by 5-layer TiO_2_ and g-C_3_N_4_/TiO_2_ thin films with different spin-coating layers. (**B**) The first-order kinetic fitting curve of the photocatalytic degradation.

The photocatalytic properties of the prepared photocatalysts were estimated by the visible-light degradation of Rh B. As shown in Figure 5A, the direct degradation of Rh B without a photocatalyst under visible light irradiation is extremely weak, which implies that the self-degradation of Rh B can be ignored. After the addition of photocatalyst, the removal rates were 5.1%, 17.9%, 31.2%, and 22.6% of Rh B under 180 min irradiation on 5-layer TiO_2_, 3-layer g-C_3_N_4_(0.5)/TiO_2_, 5-layer g-C_3_N_4_(0.5)/TiO_2_, 7-layer g-C_3_N_4_(0.5)/TiO_2_, respectively. These g-C_3_N_4_ containing-photocatalysts have much higher photocatalytic degradation efficiencies than that of pure TiO_2_ thin film, which may have resulted from the synergistic effect between g-C_3_N_4_ and TiO_2_. However, along with the increase in film layers, 7-layer g-C_3_N_4_ (0.5)/TiO_2_ has a decreased photodegradation efficiency. The kinetic rates for the photodegradation of Rh B are fitted according to the first-order reaction equation: ln(*C_0_*/*C*), where k, *C_0_,* and *C* is the rate constant, initial and real-time concentration of Rh B [24]. As shown in Figure 5B, the fitting curve of the photocatalytic degradation reaction is straight and is shown below. The k values of 5-layer TiO_2_, 3-layer g-C_3_N_4_(0.5)/TiO_2_, 5-layer g-C_3_N_4_(0.5)/TiO_2_, and 7-layer g-C_3_N_4_(0.5)/TiO_2_ are 2.90 × 10^−4^, 11.2 × 10^−4^, 21.8 × 10^−4^, and 13.3 × 10^−4^ min^−1^, respectively. Among them, 5-layer g-C_3_N_4_(0.5)/TiO_2_ shows the best photocatalytic performance and its rate constant is nearly four times higher than that of 5-layer TiO_2_. However, the photocatalytic activity decreases with the further increase in the coating layer, and 7-layer g-C_3_N_4_(0.5)/TiO_2_ demonstrates a decreased efficiency. The difference in the photocatalytic activity should come from the different characteristics of the prepared g-C_3_N_4_/TiO_2_ thin-film photocatalysts such as BET specific surface and particle size. According to the results of SEM analyses, the increase in the coating layers has an influence on the particle size and porous feature, and 5-layer g-C_3_N_4_(0.5)/TiO_2_ has the smallest particle size and most of the pores. This may help the access between the pollutant and the active sites, which finally improves the photocatalytic performance.

#### 3.3.2. Effects of Film Composition on the Photocatalytic Performance

**Figure 6:**
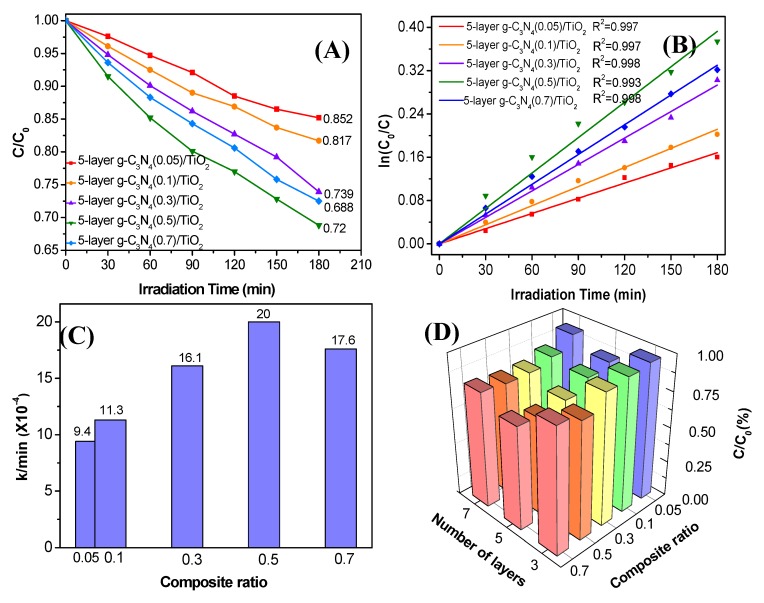
(**A**) Photocatalytic degradation of Rh B by g-C_3_N_4_/TiO_2_ thin films with a different ratio. (**B**) The first-order kinetic fitting curve of photocatalytic degradation. (**C**) The histogram of reaction constants k for Rh B degradation by g-C_3_N_4_/TiO_2_ thin films with different composition ratios under visible light. (**D**) The histogram of the three-dimensional degradation rate for all the prepared photocatalysts with different thicknesses and composition ratios.

According to the above analysis, the 5-layer thin-film photocatalyst has the highest photocatalytic activity. To further investigate the composition impact on the photocatalytic performance, 5-layer photocatalysts with different quantities of g-C_3_N_4_ were prepared and the corresponding photocatalytic degradations of Rh B were done. As seen in Figure 6A, 14.3%, 18.8%, 26.3%, 31.2%, and 28.0% of Rh B were degraded after 180 min irradiation on 5-layer g-C_3_N_4_ (0.05)/TiO_2_, 5-layer g-C_3_N_4_(0.3)/TiO_2_, 5-layer g-C_3_N_4_(0.5)/TiO_2_, and 5-layer g-C_3_N_4_(0.7)/TiO_2_, respectively. The photodegradation rate is also simulated by the above first-order kinetic fitting, as shown in Figure 6B. The photodegradation rate k for 5-layer g-C_3_N_4_(0.05)/TiO_2_, 5-layer g-C_3_N_4_(0.1)/TiO_2_, 5-layer g-C_3_N_4_(0.3)/TiO_2_, 5-layer g-C_3_N_4_(0.5)/TiO_2_, and 5-layer g-C_3_N_4_(0.7)/TiO_2_ is 9.40 × 10^−4^, 11.8 × 10^−4^, 16.3 × 10^−4^, 21.8 × 10^−4^, and 18.3 × 10^−4^·min^−1^, respectively. As the histogram in Figure 6C shows, the reaction constant K increases first and decreases afterwards with the increase in composition ratio, and the standard errors are 2.1 × 10^−5^, 2.4 × 10^−5^, 2.5 × 10^−5^, 7.1 × 10^−5^, and 3.1 × 10^−5^, respectively. It can be intuitively seen that 5-layer g-C_3_N_4_ (0.5)/TiO_2_ has the highest efficiency for Rh B degradation, which is 2.1 times more than that of 5-layer g-C_3_N_4_(0.05)/TiO_2_. This indicates that the increase in g-C_3_N_4_ can definitely improve the photocatalytic activity. However, the photocatalytic activity decreases with the further increase in g-C_3_N_4_, which might come from the reduced separation efficiency of photogenerated carriers from the self-recombination on g-C_3_N_4_. The photodegradation rates on all the prepared samples are summarized in Figure 6D. As shown, both the thickness and composition of the prepared thin film have an impact on the photocatalytic activity for Rh B degradation. The best photocatalyst could be obtained using a 5-layer coating with m(g-C_3_N_4_): m(TiO_2_) = 0.5:1.

### 3.4. Photocatalytic Stability

**Figure 7:**
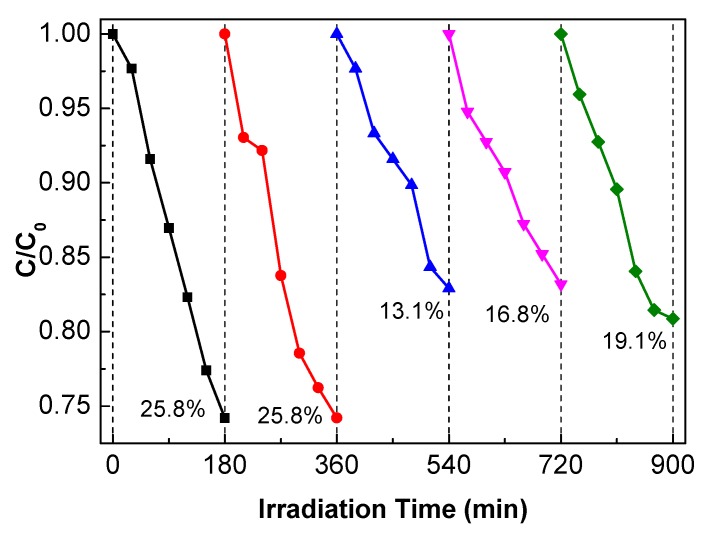
Cyclic stability test of the 5-layer g-C_3_N_4_(0.5)/TiO_2_ photocatalytic degradation of tetracycline hydrochloride (TC-HCl).

Except for the easy operation, cyclic stability is another important factor for the industrial application of thin-film photocatalysts. The antibiotic residue is another refractory pollutant in water, and the photodegradation of tetracycline hydrochloride (TC-HCl) was evaluated as the model reaction to study the photocatalytic activity as well as the stability. Five-layer g-C_3_N_4_(0.5)/TiO_2_ is selected for this evaluation and the result is shown in Figure 7. A total of 25.8% TC-HCl is degraded after 180 min of visible-light irradiation, and no reduced photocatalytic performance is observed at the 2nd cycle. However, a slight decrease in the photocatalytic activity is present after the 2nd cycle, which may come from the loss of weakly attached photocatalysts. Then, no further activity loss in the other cycles is observed, indicating that the prepared thin-film photocatalyst has stability.

### 3.5. Proposed Mechanism

**Figure 8:**
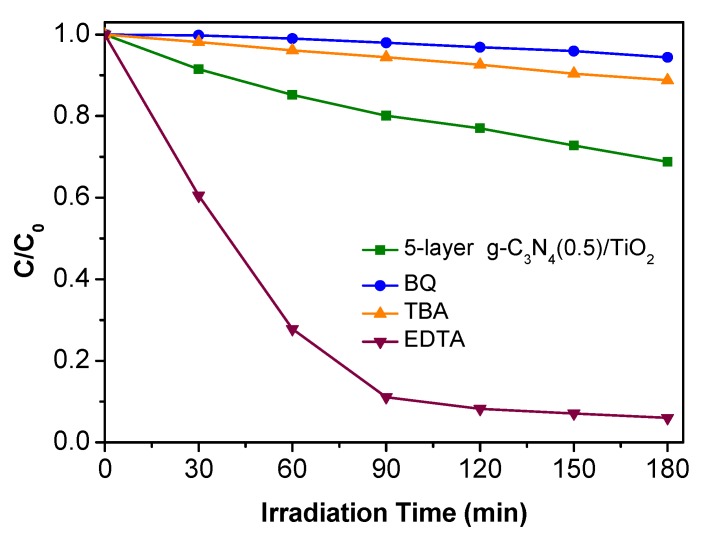
Photodegradation of Rh B over 5-layer g-C_3_N_4_(0.5)/TiO_2_ alone and with the addition of benzoquinone (BQ), tert butyl alcohol (TBA), ethylene diamine tetraacetic acid (EDTA).

The capture experiments were carried out to identify the main active substances in the photocatalytic process for understanding the corresponding reaction mechanism. Benzoquinone (BQ), tert butyl alcohol (TBA), and ethylene diamine tetraacetic acid (EDTA) were added into the Rh B solution as the trapping agent for superoxide radical (O_2_•^−^), hydroxyl radical (•OH), and hole (h^+^), respectively [29]. As shown in Figure 8, the photocatalytic effect is significantly improved after the introduction of EDTA, which suggests that h^+^ is not responsible for the photodegradation. However, the photocatalytic activity is prominently inhibited after the adding of BQ and TBA. This indicates the existence of •OH and O_2_•^−^ and they both play a vital role in the above photocatalytic degradation.

**Figure 9:**
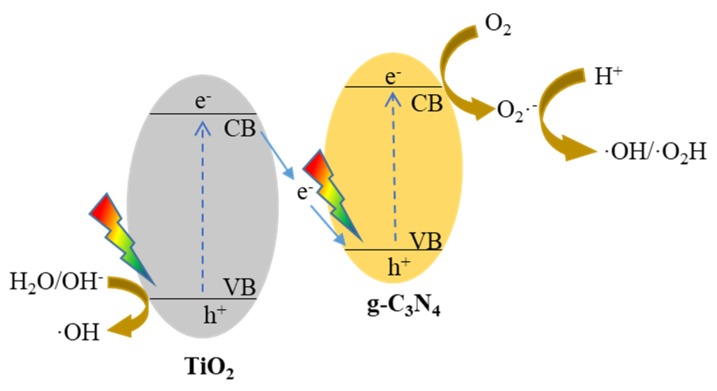
Proposed mechanism for the photodegradation of Rh B over 5-layer g-C_3_N_4_(0.5)/TiO_2_ thin film.

The reaction mechanism is discussed according to the above trapping experiment. If a traditional heterojunction is formed at the interface of 5-layer g-C_3_N_4_(0.5)/TiO_2_, photo-generated electrons may be concentrated in the conduction band of TiO_2_, and photo-generated holes are transferred into the valence band of g-C_3_N_4_. However, the valence band energy of g-C_3_N_4_ is less than the energy required for the formation of •OH, which cannot oxidize H_2_O/OH^-^ into •OH to participate in the photocatalytic reaction. Therefore, the traditional heterojunction is not responsible for the photodegradation of Rh B, and the transport of photo-generated electrons and holes in photocatalytic reactions must follow the all-solid-state Z-scheme process, which is in correspondence with previous articles [12,18]. The transport process of all-solid-state Z-scheme heterojunction carriers is shown in Figure 9. The excited electrons in TiO_2_ transfers from its conduction band to the valence band of g-C_3_N_4_. Then, the electrons are excited to the conduction band of g-C_3_N_4_ and react with the substrate to form active species. It might be pointed out that •OH is generally produced from the direct oxidation on the holes. However, it is difficult to completely exclude the formation of •OH from the consecutive reactions [30,31]. Therefore, another possible formation of •OH from the consecutive conversion of O_2_•^−^ is also given in Figure 9.

## 4. Conclusions

In summary, all-solid-state Z-scheme g-C_3_N_4_/TiO_2_ thin-film photocatalyst was successfully prepared by the facile sol-gel and spin-coating methods which showed good photocatalytic performance for the removal of colored Rh B and colorless TC-HCl with easy operation and good stability. It was found that the mass ratio of g-C_3_N_4_ to TiO_2_ and coating layers had a strong influence on the photocatalytic activity. Five-layer g-C_3_N_4_(0.5)/TiO_2_ showed the highest efficiency for the photodegradation and a Z-scheme mechanism was proposed. The construction of a thin-film photocatalyst may not only benefit the recovery from the reaction but also show good cyclic stability, which may be of great significance for practical application in the future.

## Figures and Tables

**Table 1:** The energy gaps of prepared samples.

Sample Name	Energy Gap
5-layer TiO_2_	3.7 eV
3-layer g-C_3_N_4_(0.5)/TiO_2_	3.3 eV
5-layer g-C_3_N_4_(0.5)/TiO_2_	3.25 eV
7-layer g-C_3_N_4_(0.5)/TiO_2_	3.27 eV
